# High Loading Capacity and Wear Resistance of Graphene Oxide/Organic Molecule Assembled Multilayer Film

**DOI:** 10.3389/fchem.2021.740140

**Published:** 2021-11-29

**Authors:** Li Chen, Gang Wu, Yin Huang, Changning Bai, Yuanlie Yu, Junyan Zhang

**Affiliations:** ^1^ School of Petrochemical Technology, Lanzhou University of Technology, Lanzhou, China; ^2^ Key Laboratory of Science and Technology on Wear and Protection of Materials, Lanzhou Institute of Chemical Physics, Chinese Academy of Sciences, Lanzhou, China; ^3^ Center of Materials Science and Optoelectronics Engineering, University of Chinese Academy of Sciences, Beijing, China

**Keywords:** 2D materials, self-assembly, composite films, micro/macro-tribological behaviors, high loading capacity

## Abstract

Taking advantage of the strong charge interactions between negatively charged graphene oxide (GO) sheets and positively charged poly(diallyldimethylammonium chloride) (PDDA), self-assembled multilayer films of (GO/PDDA)_n_ were created on hydroxylated silicon substrates by alternating electrostatic adsorption of GO and PDDA. The formation and structure of the films were analyzed by means of water contact angle measurement, thickness measurement, atomic force microscopy (AFM) and X-ray photoelectron spectroscopy (XPS). Meanwhile, tribological behaviors in micro- and macro- scale were investigated by AFM and a ball-on-plate tribometer, respectively. The results showed that (GO/PDDA)_n_ multilayer films exhibited excellent friction-reducing and anti-wear abilities in both micro- and macro-scale, which was ascribed to the special structure in (GO/PDDA)_n_ multilayer films, namely, a well-stacked GO–GO layered structure and an elastic 3D crystal stack in whole. Such a film structure is suitable for design molecular lubricants for MEMS and other microdevices.

## Introduction

The advances in micro-nano manufacturing technology have promoted the rapid development of microelectronic mechanical systems (MEMS) and micro/nanodevices. Due to the decrease in component size and the enhancement of surface effect, stiction, friction, and wear became significant barriers for the successful development of durable and reliable MEMS (Maboudian and Carraro, 2004; Shen and Meng, 2013). Therefore, it is very urgent to design and construct micro-nano self-lubricating system, which can improve the tribological properties between contacting surfaces on micro- and nanoscales. Molecular lubricants, because of the simple preparation method, controllable structure, high stability, and good self-recovery, are considered to be prospective candidates to resolve the tribological problems of MEMS over the last decades (Liu et al., 2012; Chen et al., 2014a; Liang et al., 2015; Cao et al., 2018). However, the inherent low loading capacity of organic molecular lubricants makes them scarcely meet the needs of long-term stability and high reliability for MEMS (Song et al., 2008; Ou et al., 2009; Chen et al., 2016). Therefore, it is desired to design and construct organic molecule films with inorganic materials such as two dimensional (2D) materials acted as steel in concrete so as to enhance the loading capacity of organic molecule films.

With excellent mechanical strength, low friction, and high chemical stability, graphene is expected to reduce the adhesion, friction, and wear problems of MEMS as a solid lubricant (Lee et al., 2008; Popov et al., 2013; Berman et al., 2014). If graphene is introduced into self-assembled films, it cannot only enhance the tribological properties of self-assembled films but also improve the loading capacity of the molecular films. However, the ideal graphene material is inert, which makes it difficult to build a powerful bond with other surfaces (Ramanathan et al., 2008; Li et al., 2019). With layer structure and some similar properties to graphene, graphene oxide (GO) and reduced graphene oxide (RGO) films may be good solid lubricants to MEMS (Mi et al., 2012; Li et al., 2013; Liang et al., 2013). GO has various active functional groups, including hydroxyl, carboxyl, and epoxy groups (Guex et al., 2017); therefore, 2D GO sheets can chemically adsorb on a variety of substrates (Mi et al., 2012; Li et al., 2013). Moreover, 2D GO sheets can be combined with organic molecular precursors to construct multilayer films *via* covalent interaction (Cassagneau et al., 2000; Ou et al., 2012; Mungse et al., 2016). Self-assembly technique is useful to prepare organic molecule films with GO. Taking advantage of the chemical reactions between epoxy/carboxyl and amine groups, GO sheets were covalently attached onto Si substrates modified by self-assembled monolayer of (3-aminopropyl) triethoxysilane (APTES SAM) (Ou et al., 2010). After thermal reduction of APTES-GO, the obtained APTES-RGO film exhibited excellent friction-reducing and anti-wear performances under low applied loads. APTES-RGO nanolayer assembled on the surface of Ti or titanium alloy substrates also holds remarkable tribological properties in nano/micro scale (Li et al., 2013; Li et al., 2019). Octadecyltrichlorosilane (OTS), a hydrophobic alkylsilane, was assembled onto the GO surface of APTES-GO layer *via* C–O–Si bonding (Ou et al., 2011). It has demonstrated that the OTS outer layer could reduce the adhesion/friction greatly, and the obtained APTES–GO–OTS multilayer film showed improved tribological performances than the APTES–GO layer. However, the loading capacity and anti-wear life of these films were still not satisfactory.

Poly(diallyldimethylammonium chloride) (PDDA), a kind of polymer with excellent stability, can be easily inserted by different methods between GO lamellae to form intercalated GO composites (Ni et al., 2010). As a novel overcoating layer for flexible transparent conductive films, this mechanically stable ultrathin film with multilayered GO and PDDA could protect silver nanowires from friction (Lee et al., 2015). Herein, 2D GO nanosheets with negative charge and PDDA with positive charge were employed to construct mechanically stable (GO/PDDA)_n_ multilayer films by a layer-by-layer (LBL) self-assembly method. In this report, we focus on the tribological behaviors of (GO/PDDA)_n_ multilayers in micro- and macro-scales. The combination of elastically strong PDDA and mechanically strong 2D GO nanosheets is expected to boost high loading capacity and anti-wear life, aiming at developing thin-film lubricants suitable for MEMS and micro/nanodevices.

## Experimental section

### Materials

**FIGURE 1:**
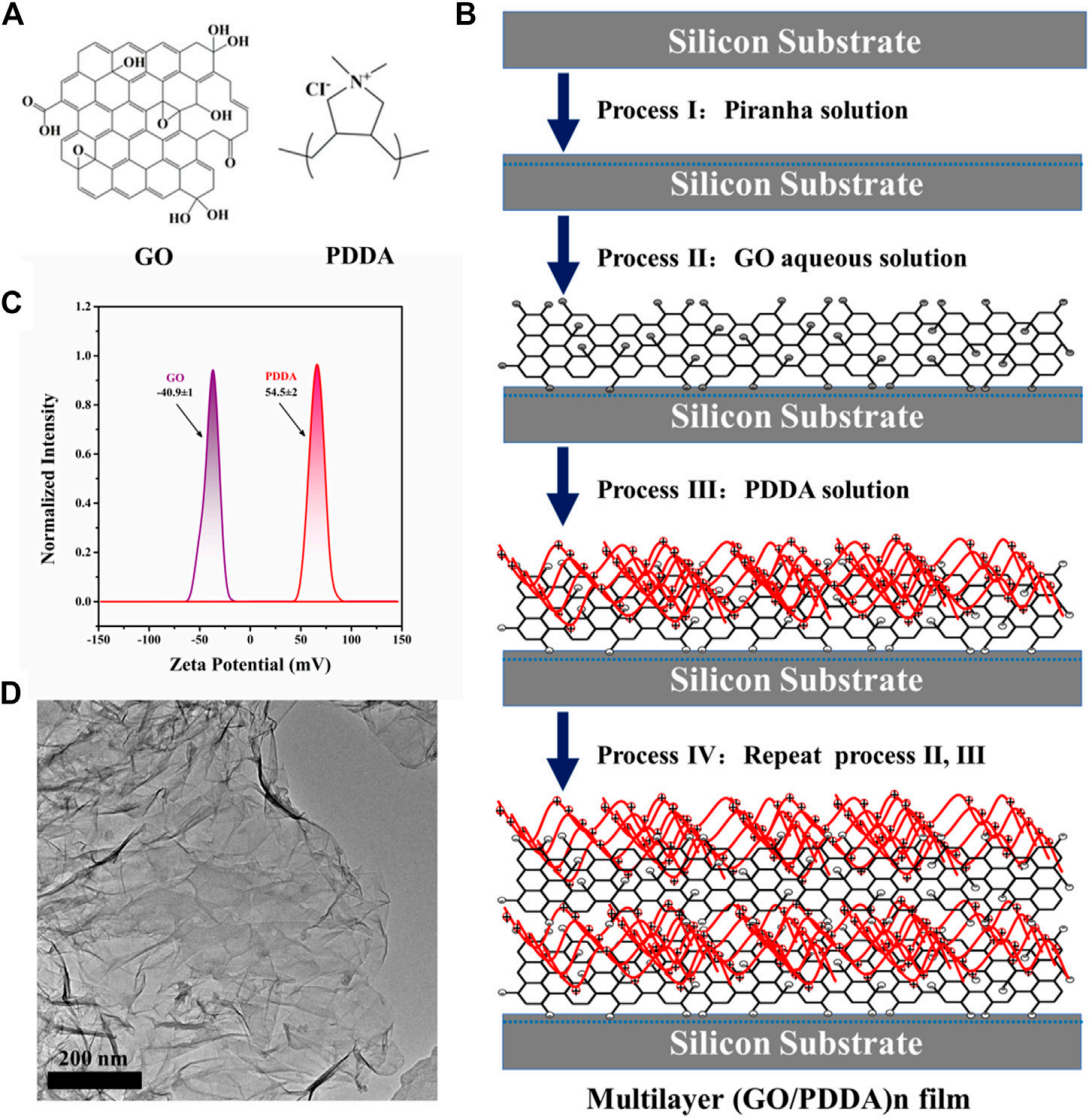
**(A)** Molecular structures of graphene oxide (GO) and poly(diallyldimethylammonium chloride) (PDDA), **(B)** Schematic illustration of the constructing process of (GO/PDDA)_n_ multilayer films by layer-by-layer (LBL) self-assembly method, **(C) ζ** potential for GO and PDDA materials, **(D)** TEM image of GO material.

Natural graphite powder was obtained from Qingdao Huatai Lubricating and Sealing Technology Co., Ltd. N-type polished single-crystal silicon (100) wafers were purchased from MCL Electronic Materials Co., Ltd. Poly(diallyldimethylammonium chloride) solution (PDDA, Mw: 100,000–200,000, 20 wt%) was purchased from Sigma-Aldrich and used as received. The chemical structures of GO and PDDA are shown in Figure 1A. Ultrapure water (>18 MΩ) was used throughout the experiment.

### Preparation of graphene oxide

Natural flake graphite powder (1.0 g) and NaNO_3_ (1.0 g) were mixed with 98% H_2_SO_4_ (50 ml) in an ice bath. KMnO_4_ (6.0 g) was slowly added to the suspension with stirring to keep the temperature below 10°C. Then the mixture was kept at 35°C and stirred for 4 h in a water bath. Subsequently, ultrapure water (100 ml) was gradually added with vigorous stirring. The reaction temperature increased rapidly and kept at 98°C for 15 min by heating. Then, the mixture was further treated with 30% H_2_O_2_ (40 ml). After cooling to room temperature, the mixture was centrifuged and washed with 5% HCl and then deionized water for several times. Water-soluble GO was obtained.

### Fabrication of self-assembled films

Silicon wafers were hydroxylated in Piranha solution (mixture of 7:3 (v/v) 98% H_2_SO_4_ and 30% H_2_O_2_) at 90°C for 30 min. After being thoroughly rinsed with ultrapure water and blown dry with N_2_, the hydroxylated silicon wafers were immersed into the GO aqueous solution at 80°C for 12 h. Then the wafers were ultrasonically cleaned in ultrapure water and blown dry with N_2_ gas, and the film was designated as GO SAM. Subsequently, GO SAM covered Si wafers were kept in the PDDA water solution (1.5 wt%) for 1 h, followed by washing with ultrapure water and drying in N_2_ gas. The obtained sample was designated as (GO/PDDA)_1_. Repeating above operation three and five times, respectively, the obtained samples were coded as (GO/PDDA)_3_ and (GO/PDDA)_5_. As a contrast, PDDA SAM was prepared by keeping hydroxylated silicon wafers in PDDA solution for 1 h and then treated using the above washing and drying steps.

### Characterization

The zeta potentials of GO and PDDA in water solution were measured with a Zetasizer Nano Series instrument (Malvern Instruments Ltd., UK) at 25°C. Water contact angles (WCA) of the samples were characterized by a contact angle meter (SZ-CAM). The reported data are average values of at least five repeated measurements for each sample. The film thicknesses were measured on a L116-E ellipsometer (Gaertner, MN, USA) equipped with a He–Ne laser (632.8 nm) at an incident angle of 50°. A refractive index of 1.46 was set for silicon oxide and 1.45 for GO, PDDA, and (GO/PDDA)_n_ layers. Raman spectra were collected by a Raman spectrometer (LabRAM HR 800, Horiba Jobin Yvon). X-ray photoelectron spectroscopy (XPS, Kratos Axis Ultra DLD) was employed to analyze the chemical composition and element chemical state on specimen surfaces. The surface morphologies of the samples were observed by a Nanoscope IIIa Multimode atomic force microscope (AFM, Digital Instruments, UK) in tapping mode.

### Friction tests

Microtribological properties of the films were investigated by a Nanoscope Шa Multimode scanning probe microscope in contact mode. V-shape Si_3_N_4_ cantilever with an announced elastic force constant of 2 N/m was used. The output voltages were directly used as the relative friction forces. The friction force–load curve was made from the friction loops. At least six separate locations on each sample were selected for measurement. The adhesive force of each specimen was an average value of at least six locations on each sample surface. All experiments were carried out under ambient conditions of 20°C and 40%–50% relative humidity.

A UMT-2MT tribometer (CETR, USA) in a ball-on-plate reciprocating mode was employed to evaluate the macrotribological performance. Si_3_N_4_ ball (*Φ* = 3 mm) used as upper counterpart was stationary, while the lower specimen adhered on the flat base kept reciprocating at a distance of 5 mm. The loads of 0.1, 0.2, 0.3, and 0.4 N were applied for the measurements. At least three repeated tests were performed for each specimen. All tests were made under ambient conditions of 20°C and 40%–50% relative humidity.

## Results and discussion

### Formation and characterization of (graphene oxide/poly(diallyldimethylammonium chloride)_n_ film

Self-assembled multilayer films of (GO/PDDA)_n_ were created on silicon wafers by alternating electrostatic adsorption of GO and PDDA, and the constructing process is shown schematically in Figure 1B. As illustrated, GO sheets were introduced onto the hydroxylated silicon wafers to create an underlayer. GO sheets can be chemisorbed onto hydroxylated silicon substrates *via* chemical reactions between hydroxyl/carboxyl and hydroxyl groups. Then negatively charged GO layer was modified by electrostatic adsorption of positively charged PDDA. The strong charge interactions sustain the GO/PDDA bilayers and maintain them during this LBL process (Qureshi et al., 2013), which was evidenced by the Figure 1C. The target film designated as (GO/PDDA)_1_, thus, was formed. Alternating electrostatic adsorption of GO sheets and PDDA three and five times, respectively, the multilayer films of (GO/PDDA)_3_ and (GO/PDDA)_5_ were obtained. It is worth noting that the preparation of the composite film is also derived from the good film-forming properties of graphene oxide, as shown in Figure 1D. Furthermore, the ability to control the thickness using the LBL method is evaluated. The film thicknesses were measured using ellipsometer (Porter et al., 1987), in which their thicknesses are, respectively 3.37, 9.41, and 15.93 nm for the (GO/PDDA)_1_, (GO/PDDA)_3_, and (GO/PDDA)_5_ layers. It can be seen that the growth of the (GO/PDDA)_n_ films was linear.

**FIGURE 2:**
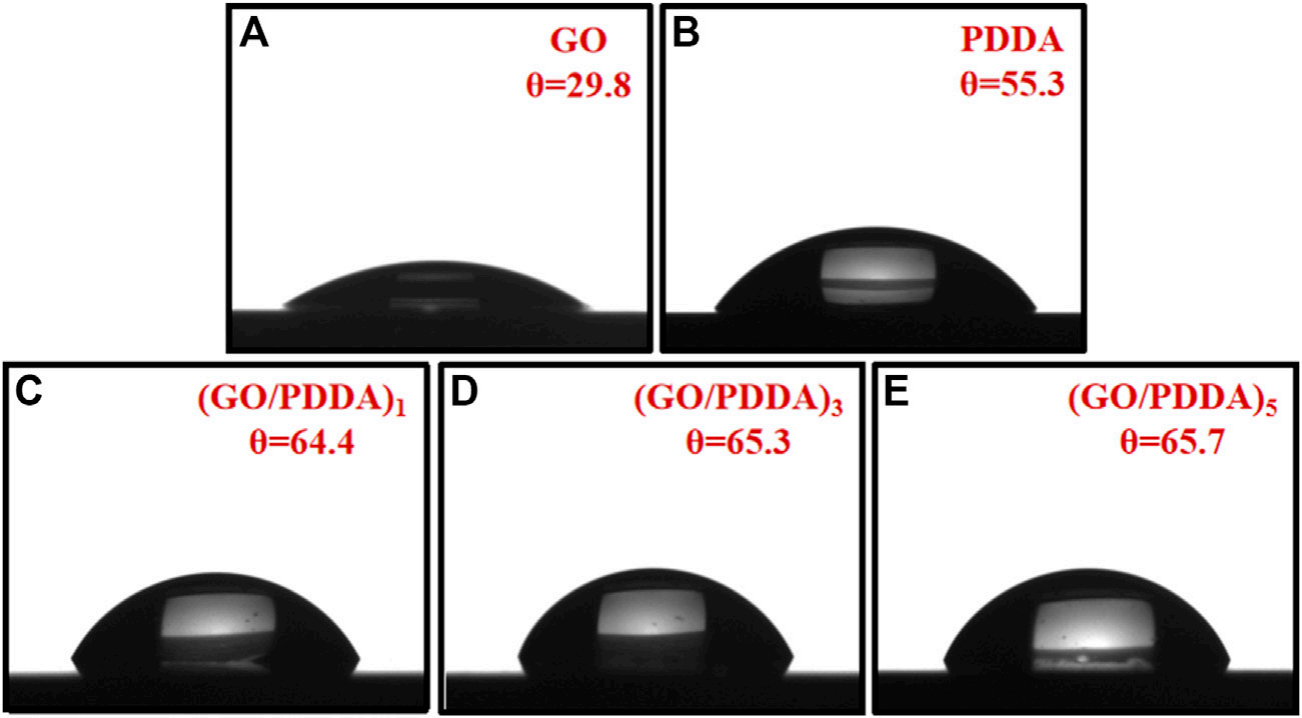
Water contact angles (WCA) of composite films.

Contact angle meter was applied to determine the surface wettability of the films, as shown in Figure 2. The WCA for silicon wafers after the hydroxylation process is less than 5°, which agrees with the reported results (Song et al., 2008; Ou et al., 2010). GO and PDDA are known as hydrophilic materials because of the oxygen-containing groups and the quaternary ammonium salt segments. The WCAs for GO SAM and PDDA SAM were 29.8° and 55.3°, respectively. The films showed similar WCA values of 64.4°, 65.3°, and 65.7° for (GO/PDDA)_1_, (GO/PDDA)_3_, and (GO/PDDA)_5_ layers, respectively. Compared with PDDA SAM, (GO/PDDA)_n_ multilayers with the topmost PDDA exhibited higher hydrophobicity. That is attributed to the strong interaction between the quaternary ammonium salt segments of PDDA and the anionic functional groups of GO, which expose the hydrophobic backbone of the topmost PDDA to the surface (Lee et al., 2015).

**FIGURE 3:**
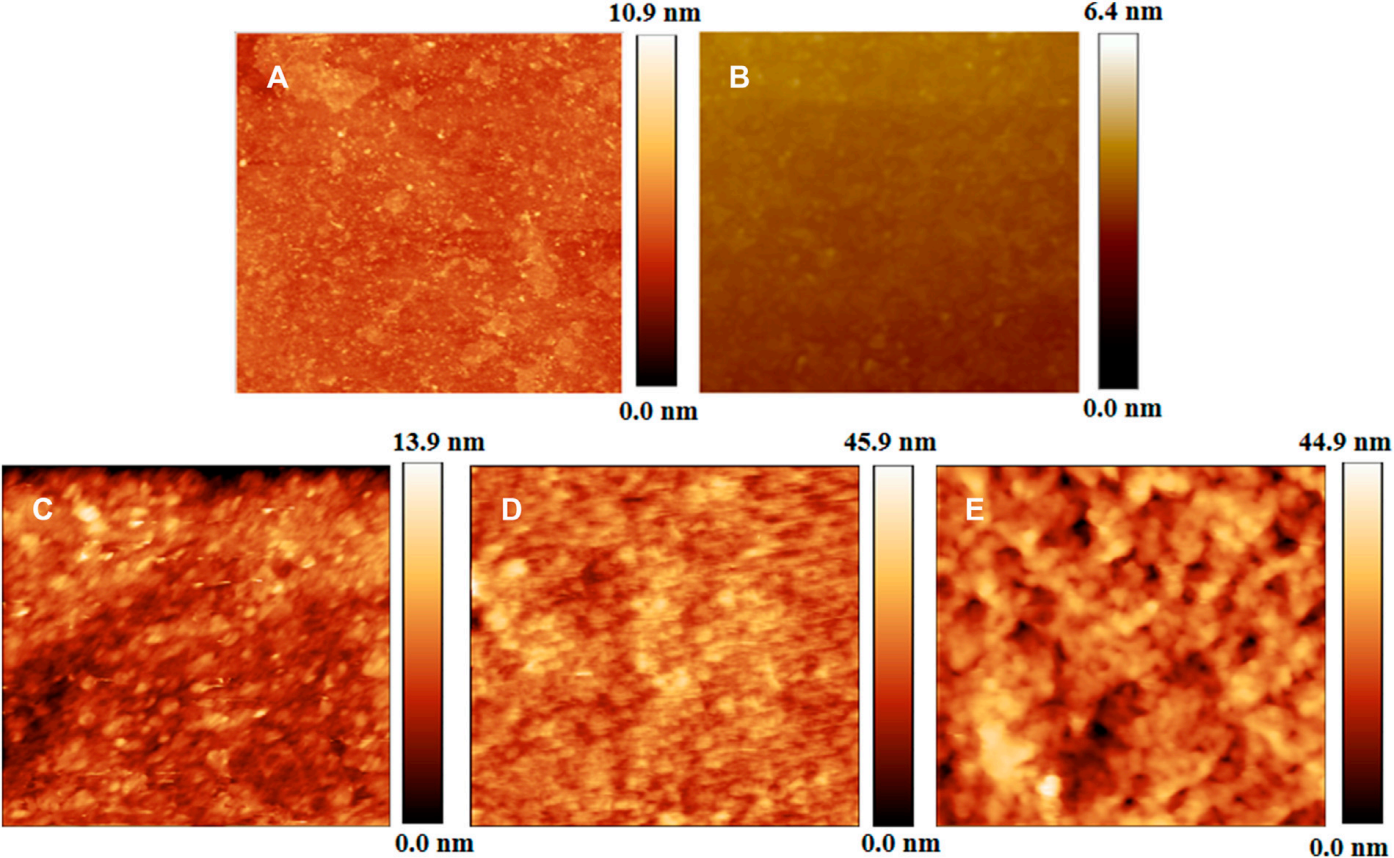
Atomic force microscope (AFM) morphologies of GO self-assembled monolayer (SAM) **(A)**, PDDA SAM **(B)**, (GO/PDDA)_1_ **(C)**, (GO/PDDA)_3_ **(D)**, and (GO/PDDA)_5_ **(E)** over a scanning area of 2.5 μm × 2.5 μm.

The AFM morphology in Figure 3A shows that the irregular GO sheets were discontinuously distributed on Si substrate. PDDA SAM is relatively smooth with the root-mean-square (RMS) microroughness of about 1.29 nm over a scanning range of 2.5 μm × 2.5 μm, while the (GO/PDDA)_n_ films are composed of plentiful regular grains, and the surfaces are relatively rough with the RMS microroughnesses of about 1.76, 3.04, and 4.70 nm for the (GO/PDDA)_1_, (GO/PDDA)_3_, and (GO/PDDA)_5_ layers, respectively.

**FIGURE 4:**
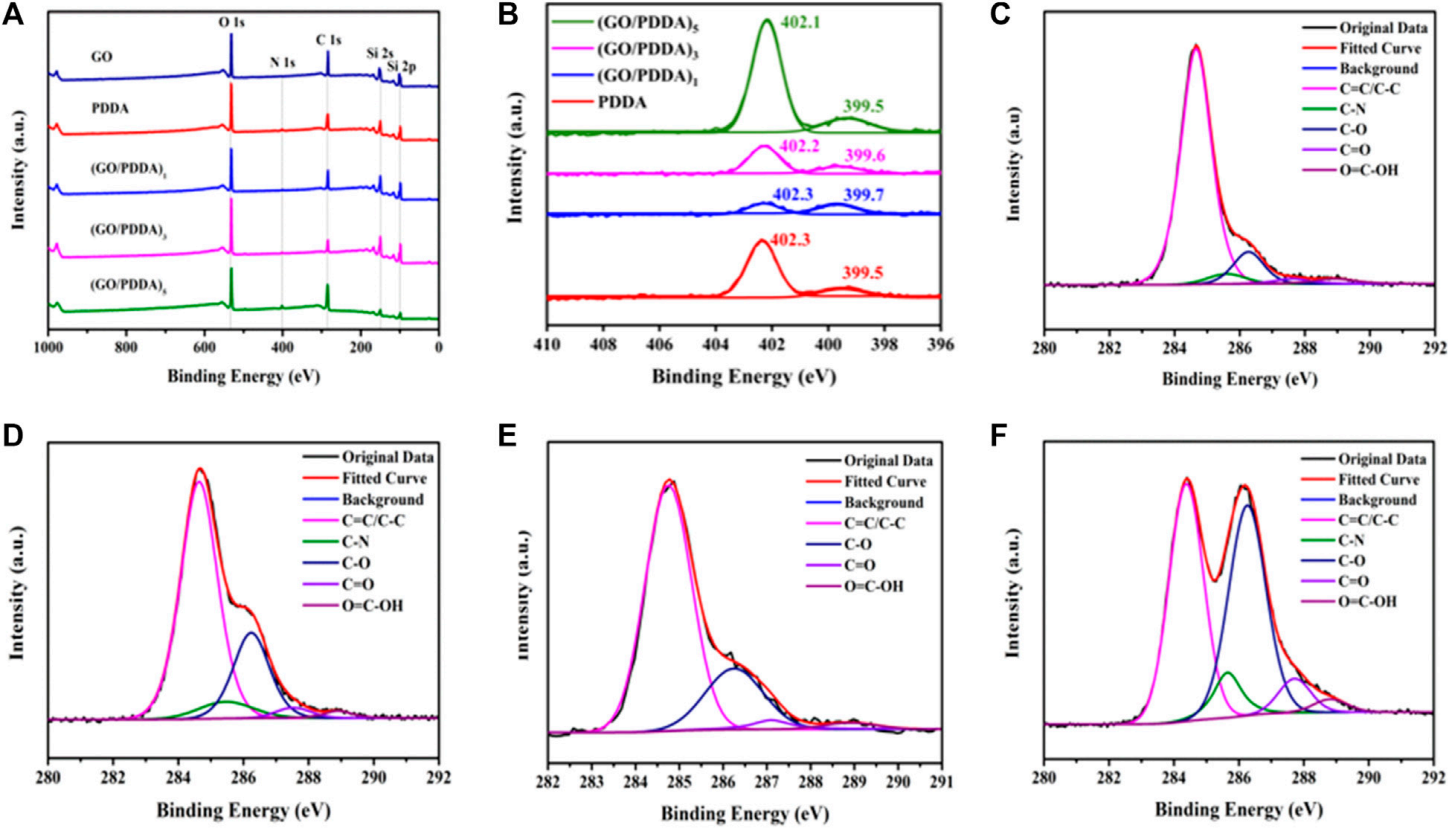
X-ray photoelectron spectroscopy (XPS) survey spectra for different samples **(A)**, N 1s spectra for PDDA film and (GO/PDDA)_n_ films **(B)**, C 1s spectra for GO material **(C)**, (GO/PDDA)_1_ **(D)**, (GO/PDDA)_3_ **(E)**, (GO/PDDA)_5_ **(F)**.

XPS was employed to analyze the detailed composition and the element chemical status of the as-prepared films (Figure 4). The N 1s peak at 400.1 eV in the spectrum of PDDA indicated that PDDA was successfully assembled on silicon surface (Figures 4A, B). In all spectra of (GO/PDDA)_n_ films, the N 1s signals centered at 400.1 eV indicated the successful covalent attachment of PDDA on GO layer. The fine spectrum of C 1s region of GO layer (Figure 4C) was deconvoluted into C=C/C–C, C–O, C=O, and O=C-OH bands at 284.8, 286.3, 287.1, and 288.9 eV, respectively (Yang et al., 2009; Stankovich et al., 2007; Tang et al., 2009). C 1s spectrum of (GO/PDDA)_1_ film in Figure 4D could be fitted to five bands at 284.7, 285.6, 286.3, 287.6, and 288.9 eV, which were attributed to C=C/C–C, C–N, C–O, C=O, and O=C–OH groups, respectively. C 1s spectra of (GO/PDDA)_3_ and (GO/PDDA)_5_ films (Figures 4E, F) could be deconvoluted into the same bands as that of (GO/PDDA)_1_ film. The content of C–N from the C 1s fitting results was 4.11%, 6.66%, and 7.30% for (GO/PDDA)_1_, (GO/PDDA)_3,_ and (GO/PDDA)_5_ films, respectively. The content of C-N increases with the increasing number of (GO-PDDA)_n_ layers, which indicated that PDDA and GO could form an electrostatic interaction (Lee et al., 2015) in (GO/PDDA)_n_ films.

**FIGURE 5:**
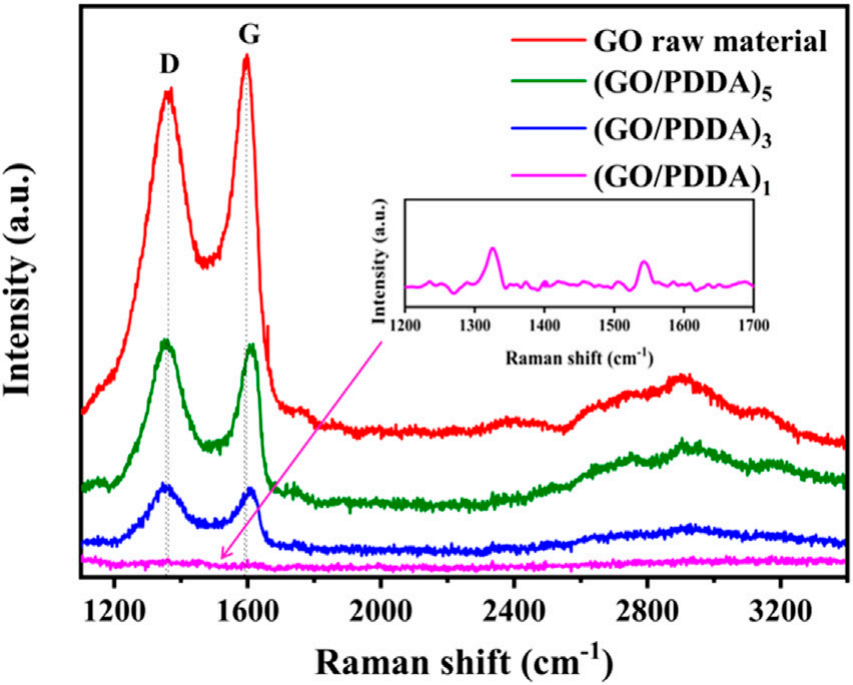
Raman spectra of GO material and (GO-PDDA)_n_ films.

Figure 5 shows the Raman spectra of GO and (GO/PDDA)_n_ films. Clearly the D peak that appeared at ∼1,350 cm^−1^ and the G peak that appeared at ∼1,590 cm^−1^ are the primary features of the Raman spectrum for GO sheets (Tan et al., 2004). The D and G peaks for the (GO/PDDA)_1_ layer were almost invisible, while the (GO/PDDA)_3_ and (GO/PDDA)_5_ layers exhibited clear D and G peaks at about 1,355 and 1,605 cm^−1^, respectively. As the number of (GO-PDDA)_n_ layers increases, the intensity of the D and G bands increased, and the peaks of the D and G bands became sharper. It means that a well-stacked GO–GO-layered structure was still maintained in (GO/PDDA)_n_ layer (Ni et al., 2010; Lee et al., 2015). Moreover, the sliding phenomenon between the GO sheets separated by PDDA appears to dominate as increasing (GO/PDDA)_n_ layers (Lee et al., 2015).

### Microtribological behaviors

**FIGURE 6:**
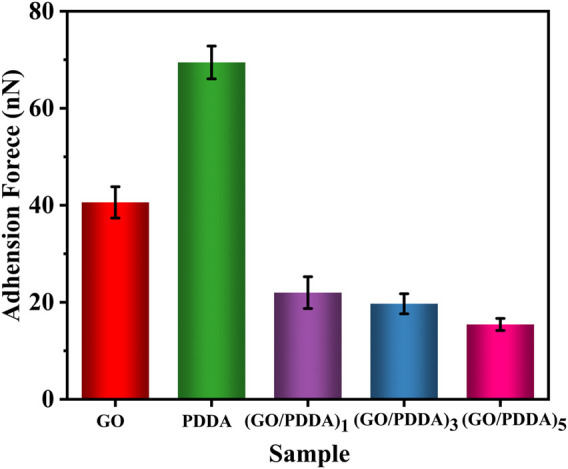
Adhesive forces for different samples.

Figure 6 presents the adhesive forces between the AFM tip and the sample surfaces. Strong adhesions were observed on the surfaces of GO layer and PDDA SAM. The adhesive force is closely related to the water contact angle (Figure 2). The surface with higher hydrophobicity exhibits lower adhesion because the adhesion is mainly dominated by capillary force between the AFM tip and the sample surface (Ou et al., 2009). Compared with PDDA SAM, (GO/PDDA)_n_ multilayers possessed the better adhesion-resistance performance. Moreover, the (GO/PDDA)_n_ films with the topmost PDDA showed similar adhesive forces.

**FIGURE 7:**
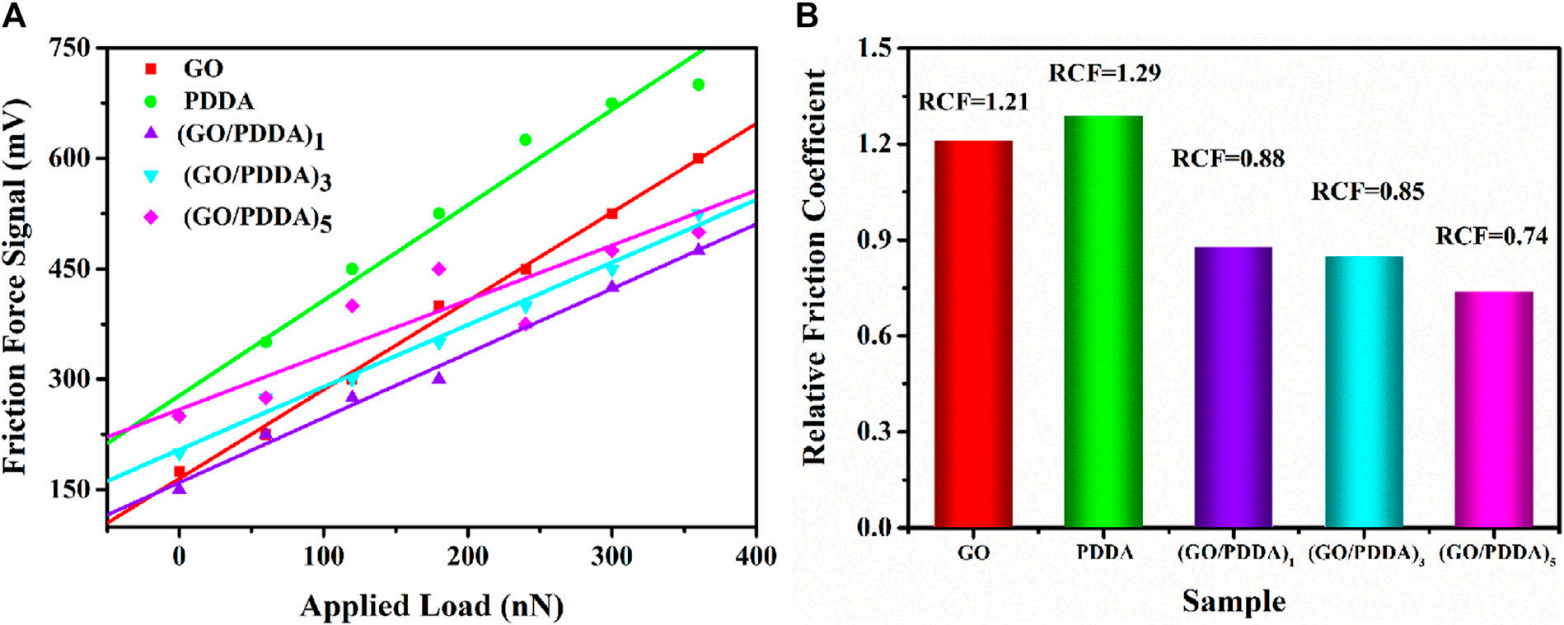
**(A)** Friction force versus load curves and **(B)** relative friction coefficient for different samples.

The cantilever deflection voltage caused by friction is proportional to the actual friction force in AFM testing, so the output voltage signal is used as the friction force (Figure 7A). In general, adhesive force is an important factor in controlling the friction behavior. Decreasing adhesion is conducive to reducing friction forces of self-assembled films on microscale (Ou et al., 2011; Chen et al., 2014b; Chen et al., 2016). (GO/PDDA)_n_ films with the topmost PDDA showed lower friction force than PDDA SAM due to the higher hydrophobicity and lower adhesive force of (GO/PDDA)_n_ films. With the increasing number of (GO/PDDA)_n_ layers, (GO/PDDA)_n_ films exhibited higher friction force because of the larger roughness. Moreover, the profiles of friction force versus external loads are approximately linear, which can be well fitted by Amonton’s law:

$$F_{L}=\mu F_{N}+F_{0}$$ (1)

where *F*_L_ is the friction force, *μ* is friction coefficient, *F*_N_ is the normal force, and *F*_0_ is the friction force when the normal force is zero (Chen et al., 2014b; Foster et al., 2006; Houston et al., 2005). It is clearly obtained that both GO SAM and PDDA SAM possess high relative friction coefficients, while the (GO/PDDA)_n_ multilayer films exhibit much better lubricity (Figure 7B). It can be attributed to the higher hydrophobicity of the (GO/PDDA)_n_ multilayer films, which plays an important part in reducing adhesive force between AFM tip and film surface. Many researchers have found that the friction would decrease with the reducing adhesion (Houston et al., 2005; Foster et al., 2006; Ou et al., 2011; Chen et al., 2014b; Chen et al., 2016).

### Macrotribological behaviors

**FIGURE 8:**
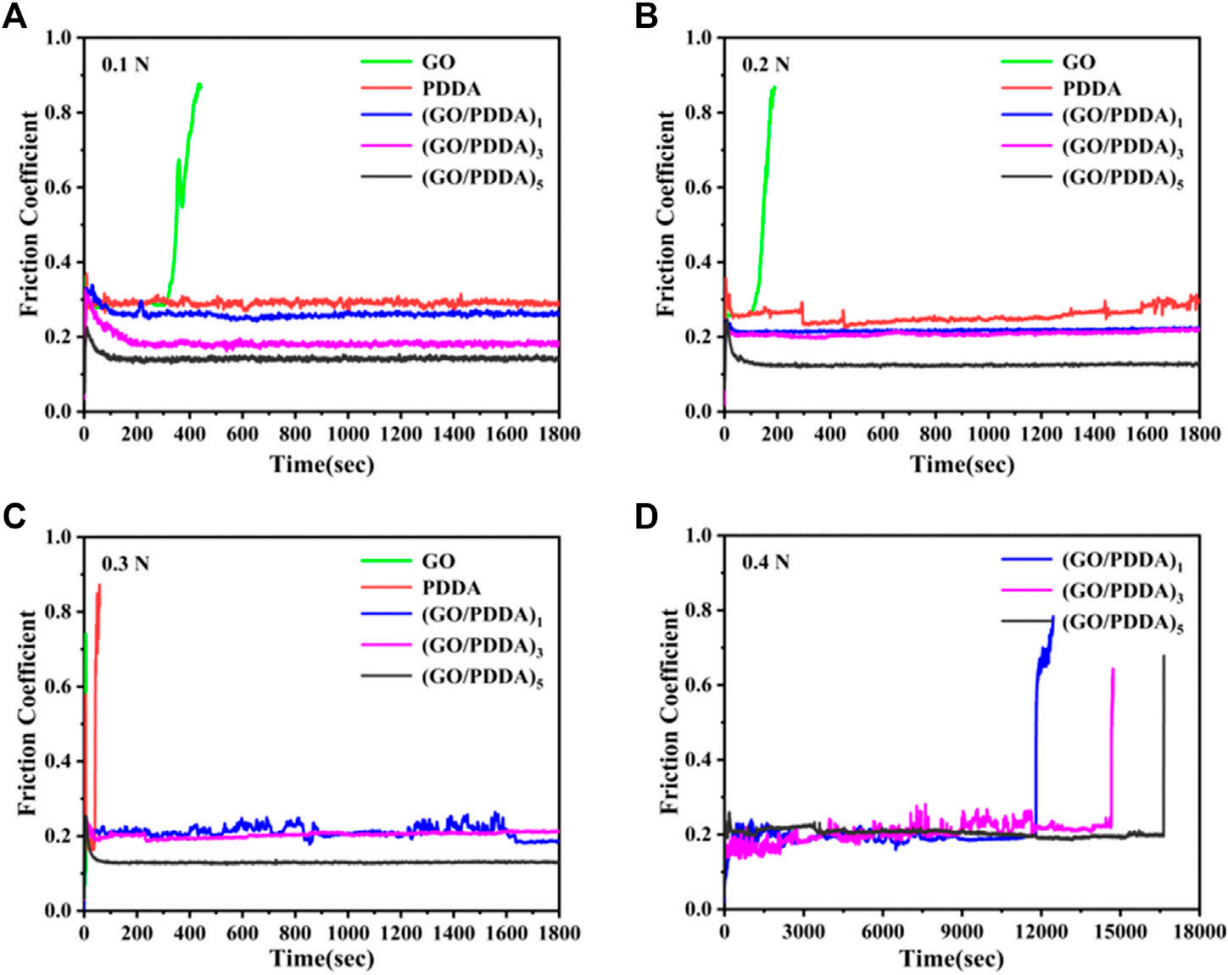
Variation in friction coefficient with time for various sample at different applied loads and a sliding frequency of 1 Hz: **(A)** 0.1 N; **(B)** 0.2 N; **(C)** 0.3 N; **(D)** 0.4 N.

The wear resistance is an important factor for lubricant films. The macrotribological behaviors of the prepared films were evaluated by a ball-on-plate tribometer, and the results are shown in Figure 8. The GO layer showed poor tribological properties and very short anti-wear life. For PDDA SAM, the friction coefficient is about 0.3 under the loads of 0.1 and 0.2 N (Figures 8A, B). PDDA SAM was worn out quickly under the load of 0.3 N (Figure 8C). It means that the load-carry capacity of PDDA SAM is poor. In contrast, (GO/PDDA)_n_ multilayer films exhibited excellent friction-reducing and anti-wear abilities. The friction coefficients of (GO/PDDA)_n_ films are lower than that of PDDA SAM in all the applied loads, and the friction coefficients of multilayer films show a gradual decrease as the increasing number of (GO/PDDA)_n_ layers. Besides, the (GO/PDDA)_n_ films possessed higher load-carrying capacity. Under the conditions of 0.4 N and 1 Hz, they showed anti-wear life of more than 11,800, 14,600, and 16,600 s for the (GO/PDDA)_1_, (GO/PDDA)_3_, and (GO/PDDA)_5_ layers, respectively (Figure 8D). The excellent lubricity can be attributed to the special structure in (GO/PDDA)_n_ multilayer films. PDDA enlarge GO sheets to fabricate an elastic 3D crystal stack rather than a rigid 3D crystal stack composed of only GO sheets (Lee et al., 2015), which is a benefit to improve the anti-wear and load-carrying properties. Besides, a well-stacked GO–GO-layered structure was still maintained in (GO/PDDA)_n_ layer (as mentioned in Raman analysis, Figure 5). The sliding phenomenon between the GO sheets is conducive to reducing friction coefficient. The sliding phenomenon appears to dominate as the number of (GO/PDDA)_n_ layers increases, which causes a decrease in friction coefficient with increasing number of (GO-PDDA)_n_ layers.

## Conclusion

(GO/PDDA)_n_ multilayer films with the topmost PDDA were constructed on hydroxylated silicon substrates by a LBL self-assembly method, where negatively charged GO sheets and positively charged PDDA were adsorbed alternately. The relationship between the microstructures and tribological behaviors of the films was studied. The results indicated that (GO/PDDA)_n_ multilayer films exhibits excellent tribological behaviors at micro- and macroscale. Moreover, the film possesses better friction-reducing and anti-wear abilities with increasing number of (GO-PDDA)_n_ layers. It could be attributed to the special structure in (GO/PDDA)_n_ multilayer films. PDDA enlarge GO sheets to fabricate an elastic 3D crystal stack, which is a benefit to improve the anti-wear and load-carrying properties. Besides, a well-stacked GO–GO-layered structure was still maintained in the (GO/PDDA)_n_ layer, which is conducive to reducing friction coefficient, and the microstructure enhanced with increasing number of (GO-PDDA)_n_ layers. Therefore, such film structure is suitable to construct lubricant coatings for MEMS and other microdevices.

## Data Availability

The original contributions presented in the study are included in the article/Supplementary Material. Further inquiries can be directed to the corresponding author.
